# Estrogen Receptor Alpha Expression in Podocytes Mediates Protection against Apoptosis *In-Vitro* and *In-Vivo*

**DOI:** 10.1371/journal.pone.0027457

**Published:** 2011-11-11

**Authors:** Sebastian Kummer, Stefanie Jeruschke, Lara Vanessa Wegerich, Andrea Peters, Petra Lehmann, Annette Seibt, Friederike Mueller, Nadezda Koleganova, Elisabeth Halbenz, Claus Peter Schmitt, Markus Bettendorf, Ertan Mayatepek, Marie-Luise Gross-Weissmann, Jun Oh

**Affiliations:** 1 Department of General Pediatrics and Neonatology, University Children's Hospital Duesseldorf, Duesseldorf, Germany; 2 Pediatric Nephrology, Pediatrics II, University of Duisburg-Essen, Essen, Germany; 3 Institute of Pathology, University Hospital Heidelberg, Heidelberg, Germany; 4 Division of Pediatric Endocrinology and Diabetology, University Children's Hospital, Heidelberg, Germany; 5 Division of Pediatric Nephrology, University Children's Hospital, Heidelberg, Germany; 6 Department of Pediatric Nephrology, University Medical Center, Hamburg-Eppendorf, Hamburg, Germany; University of Houston, United States of America

## Abstract

**Context/Objective:**

Epidemiological studies have demonstrated that women have a significantly better prognosis in chronic renal diseases compared to men. This suggests critical influences of gender hormones on glomerular structure and function. We examined potential direct protective effects of estradiol on podocytes.

**Methods:**

Expression of estrogen receptor alpha (ERα) was examined in podocytes *in vitro* and *in vivo*. Receptor localization was shown using Western blot of separated nuclear and cytoplasmatic protein fractions. Podocytes were treated with Puromycin aminonucleoside (PAN, apoptosis induction), estradiol, or both in combination. Apoptotic cells were detected with *Hoechst* nuclear staining and Annexin-FITC flow cytometry. To visualize mitochondrial membrane potential depolarization as an indicator for apoptosis, cells were stained with tetramethyl rhodamine methylester (TMRM). Estradiol-induced phosphorylation of ERK1/2 and p38 MAPK was examined by Western blot. Glomeruli of ERα knock-out mice and wild-type controls were analysed by histomorphometry and immunohistochemistry.

**Results:**

ERα was consistently expressed in human and murine podocytes. Estradiol stimulated ERα protein expression, reduced PAN-induced apoptosis *in vitro* by 26.5±24.6% or 56.6±5.9% (flow cytometry or *Hoechst*-staining, respectively; both p<0.05), and restored PAN-induced mitochondrial membrane potential depolarization. Estradiol enhanced ERK1/2 phosphorylation. In ERα knockout mice, podocyte number was reduced compared to controls (female/male: 80/86 vs. 132/135 podocytes per glomerulus, p<0.05). Podocyte volume was enhanced in ERα knockout mice (female/male: 429/371 µm^3^ vs. 264/223 µm^3^ in controls, p<0.05). Tgfβ1 and collagen type IV expression were increased in knockout mice, indicating glomerular damage.

**Conclusions:**

Podocytes express ERα, whose activation leads to a significant protection against experimentally induced apoptosis. Possible underlying mechanisms include stabilization of mitochondrial membrane potential and activation of MAPK signalling. Characteristic morphological changes indicating glomerulopathy in ERα knock-out mice support the *in vivo* relevance of the ERα for podocyte viability and function. Thus, our findings provide a novel model for the protective influence of female gender on chronic glomerular diseases.

## Introduction

Numerous epidemiological and animal studies demonstrated that women have a significantly better renal outcome in chronic glomerular diseases compared to men [1], [2], [3], [4]. During the physiological aging process, glomerular filtration rate (GFR) declines faster in males than in females between 20 and 50 years of age [5].

Lifestyle factors such as nutrition, smoking, and cardiovascular risk factors (e.g. arterial hypertension) were identified as being critical for a better renal prognosis in women. Nevertheless, these do not fully explain the gender differences seen in various kidney diseases, as several studies adjusted for these risk factors have shown [3], [6].

In animal models, renal function is influenced by gender. Aging male rats spontaneously develop proteinuria and glomerulosclerosis, whereas female animals are remarkably resistant to these changes [7]. These sequelae are largely prevented by estrogen treatment alone [8] or in combination with orchiectomy [9] in males. Female ERα knockout (KO) mice develop albuminuria, glomerular hypertrophy and glomerular sclerosis between 6 and 9 months of age [10], [11], compensatory kidney hypertrophy is reduced following unilateral nephrectomy [12]. In other experimental models of renal damage, such as uninephrectomy and ovarectomy of spontaneously hypertensive rats (SHRsp) or Puromycin aminonucleoside (PAN)-induced nephrosis, estradiol reduced the expression of different glomerular damage markers [13], [14].

Reduced podocyte number, e.g. by podocyte apoptosis, is critical for the development of proteinuria, glomerulosclerosis and progressive kidney failure [15], [16]. Consequently, apoptosis is regarded as one of the key factors in multiple glomerular diseases, especially focal-segmental glomerulosclerosis (FSGS) [17]. In different non-renal cell types, numerous articles have shown that gender hormones, in particular estrogens, have direct influences on apoptosis through the binding to estrogen receptors (ER) [18]. Cytoplasmic and nuclear ER induce transcriptional regulation of genes encoding for mitochondrial proteins, which indicates a link between ER signalling and intact mitochondrial function [19]. Non-classical actions via membrane-associated estrogen receptors include activation of multiple cytoplasmic signalling pathways [20]. These result in protein modification without any genomic action (e.g. phosphorylation processes), and in indirect genomic effects via downstream signalling cascades modifying gene transcription. Activation of mitogen-activated protein kinase (MAPK) pathway, for example, occurs within minutes of estrogen administration [21]. It comprises three major families of intracellular signalling molecules (extracellular signal-regulated kinase (ERK1/2), p38 MAPK, and c-Jun N-terminal kinases) with downstream effects on cell proliferation, differentiation, motility, survival, and apoptosis [22].

Both types of signalling pathways – transcriptional regulation via nuclear ER and regulation of phosphorylation cascades via membranous and cytoplasmic ER – are able to protect cells against apoptotic stimuli.

Regarding the crucial role of podocytes for chronic glomerular diseases, we investigated expression of ERα on podocytes *in-vitro* and *in-vivo*. Furthermore, we examined possible protective actions of estradiol treatment on experimentally induced apoptosis in cultured podocytes. One essential mechanism involved in apoptosis is destabilization of mitochondrial function, which we visualized by staining mitochondrial membrane potential.

Finally, we correlated the *in-vivo* findings with podocyte number and morphology, and markers of glomerular damage in ERα knockout mice compared to wild-type and heterozygous controls.

## Results

### ERα is expressed in cultured murine podocytes, mouse and human kidney tissue

**Figure 1:**
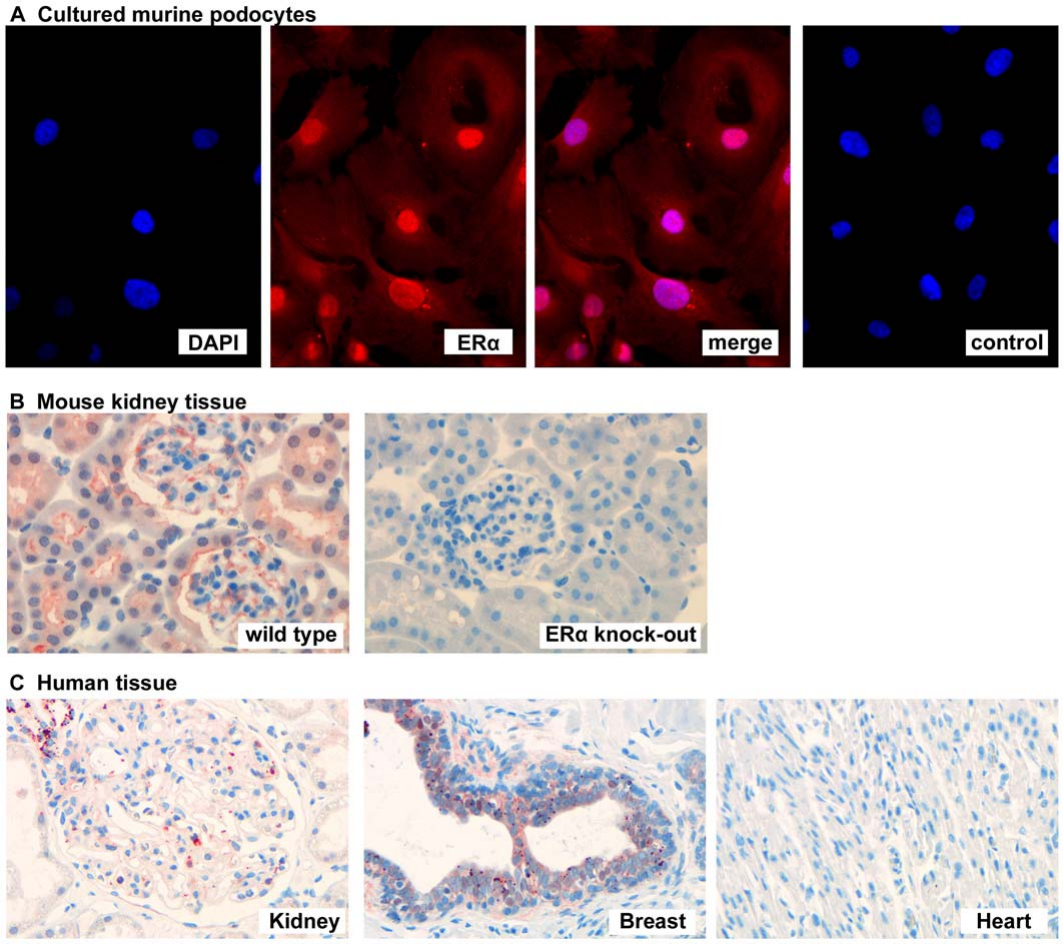
Expression of ERα in podocytes. A) Immunocytochemical staining of ERα (red) in cultured murine podocytes with cytoplasmatic and nuclear localization. DAPI was used for nuclear staining (blue), negative control was performed without ERα primary antibody. B) Immunohistochemical detection of ERα protein (red) in mouse kidney. ERα KO mice completely lack ERα expression in kidney tissue. C) Immunohistochemical detection of ERα protein (red) in human kidney tissue. Breast tissue and heart tissue were used as positive and negative controls, respectively.

Immunocytochemical stainings of cultured murine podocytes showed ERα protein with both cytoplasmic and nuclear staining (Fig. 1A).

In wild-type mouse kidney, ERα protein was found in tubular and glomerular cells, while expression was absent in ERα KO mice (Fig. 1B). Immunohistochemical staining of human renal biopsies showed glomerular expression of ERα (Fig. 1C).

**Figure 2:**
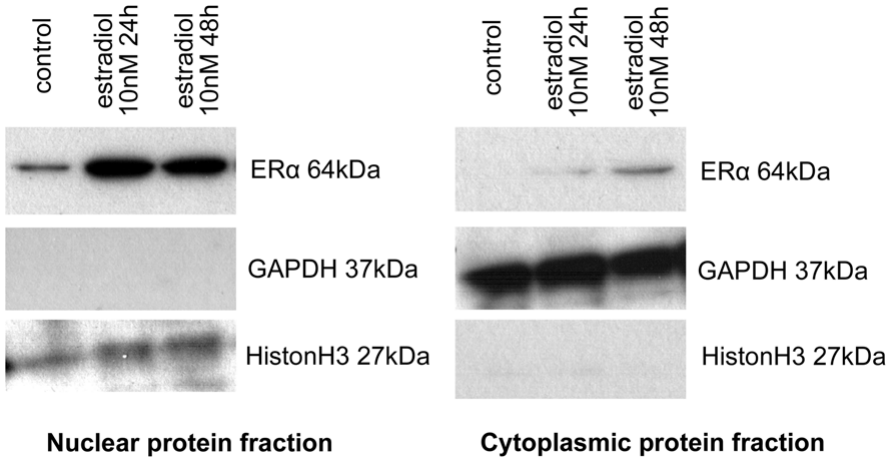
Western blot of ERα protein in cultured podocytes. In control cells without any residual estrogenic influence, the receptor protein was detected only in nuclear protein fraction. After stimulation with 10 nM estradiol for 24 h, ERα protein increased significantly in the nucleus, but was also detected in the cytoplasmatic protein fraction. Prolonged stimulation with estradiol for 48 h further increased the amount of ERα protein in the cytoplasm, while nuclear protein remained unchanged.

Western blot analyses confirmed the presence of ERα protein in murine podocytes (Fig. 2). In control cells without any residual estrogenic influence, significant amount of receptor protein was detected only in the nuclear protein fraction. After stimulation with 10nM estradiol for 24h, ERα protein increased significantly in the nucleus, but became also detectable in the cytoplasmic protein fraction. Prolonged stimulation with estradiol for 48h further increased the amount of ERα protein in the cytoplasm, while nuclear protein remained unchanged.

### Estradiol protects podocytes from apoptosis

**Figure 3:**
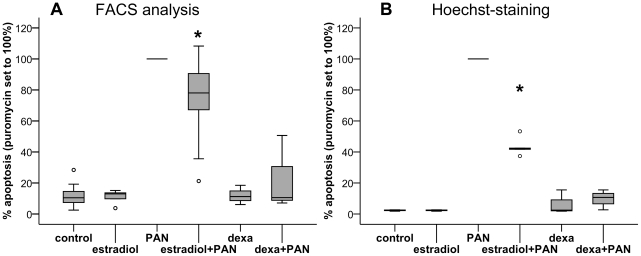
Apoptosis quantification. A) FACS-analysis of apoptosis in murine podocytes. Combination of PAN with estradiol reduced apoptosis by an average of 26.5% compared to PAN treatment alone (* p = 0.014). Dexamethasone was used as a positive control. B) Hoechst nuclear staining for the detection of apoptosis in murine cultured podocytes. Combination of PAN with estradiol reduced apoptosis by an average of 56.6% compared to PAN treatment alone (* p<0.001).

PAN treatment of cultured murine podocytes led to a significant induction of apoptosis. The amount of apoptosis in the puromycin-treated group varied between 7.9% and 36.5% (FACS-analysis), or 18.6% and 27.3% (*Hoechst*-staining), respectively (Fig. 3). Variations within one experiment were negligible. Apoptosis after PAN treatment was set to 100% in each experiment for normalization, other treatment groups are given as a percentage relative to PAN-treatment.

Analyzed by flow cytometry, estradiol treatment, which started 24 h prior to the start of incubation with PAN, induced a significant reduction of apoptosis compared to PAN treatment alone, namely by an average of 26.5±24.6% (p = 0.014, Fig. 3A). Even more effective protection against PAN-induced damage was achieved using dexamethasone, which was included as a positive control (77±24.2% average reduction in apoptosis compared to PAN treatment alone, p = 0.001).

*Hoechst* nuclear staining showed similar results: Combining PAN-treatment with estradiol led to a 56.6±5.9% reduction of apoptosis compared to PAN treatment alone (p<0.001, Fig. 3B). Dexamethasone reduced PAN-induced apoptosis by 90.1±5.3% (p = 0.001).

### Estradiol stabilizes mitochondrial membrane potential

**Figure 4:**
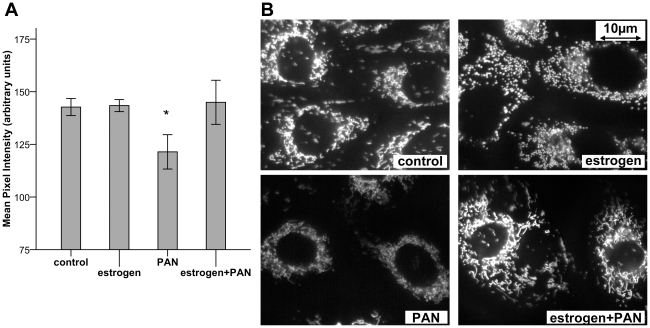
TMRM staining of mitochondrial membrane potential. A) TMRM staining shows reduced mean pixel intensity after PAN treatment, indicating loss of mitochondrial membrane potential. This can be fully prevented by co-treatment with estradiol (*p = 0.026). B) Representative images of TMRM staining.

PAN treatment induced a significant loss of TMRM staining intensity within 48 hours (121.4±4.1 arbitrary units, control: 142.7±2.0 arbitrary units; p = 0.026; Fig. 4). This finding indicates a depolarization of mitochondrial membrane potential, an initial step of the apoptotic process. Combination of PAN with estradiol prevented the loss of mitochondrial membrane potential compared to PAN treatment alone (146.1±3.9 vs. 121.4±4.1 arbitrary units; p = 0.026).

### Estradiol induces anti-apoptotic MAPK signalling by phosphorylation of ERK1/2

**Figure 5:**
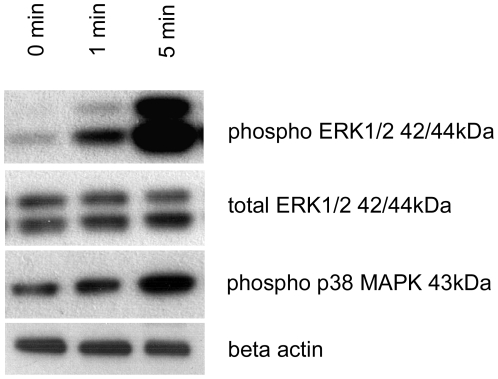
MAPK phosphorylation after estrogen treatment. Estradiol extensively stimulated ERK1/2 phosphorylation, beginning already 1 min after beginning of treatment (Fig. 5). In contrast, we detected only very small stimulation of p38 MAPK phosphorylation.

Estradiol extensively stimulated ERK1/2 phosphorylation, beginning already 1 min after beginning of treatment (Fig. 5). In contrast, we detected only very small stimulation of p38 MAPK phosphorylation.

### Reduced podocyte number, increased podocyte volume and increased TGFβ1 and collagen Type IV expression in ERα knockout mice

**Figure 6:**
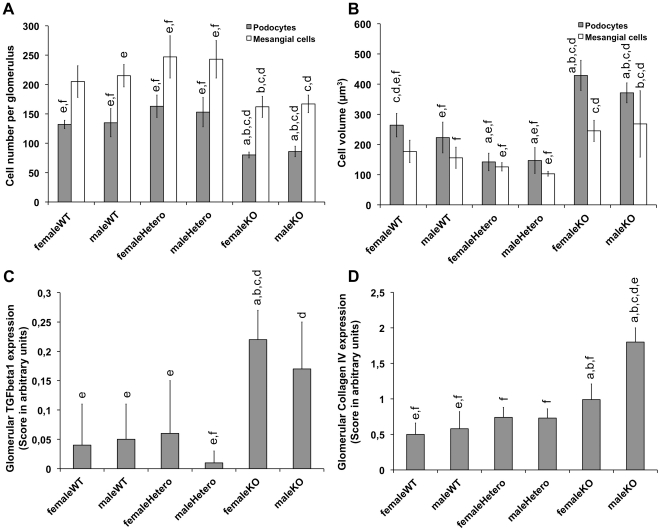
Glomerular cells and matrix in ERα knockout mice. Compared to heterozygeous and wild-type animals, podocyte numbers were significantly reduced (A) and podocyte volume significantly increased (B) in ERα knockout animals. Mesangial cell number and volume showed the same tendencies, but reached significance only in some of the intergroup comparisons (A, B). Tgfβ1 and Collagen type IV expression is increased in KO animals (C, D). n = 6 animals per treatment group. ^a^ p<0.05 vs. femaleWT, ^b^ p<0.05 vs. maleWT, ^c^ p<0.05 vs. femaleHetero, ^d^ p<0.05 vs. maleHetero, ^e^ p<0.05 vs. femaleKO, ^f^ p<0.05 vs. maleKO. ANOVA with Tamhane post-hoc analysis (p<0.01).

Six-month old ERα knockout mice did not show any significant differences compared to wild-type and heterozygous mice regarding glomerular sclerosis index, number of glomeruli and mean glomerular volume (Table 1). Total number of podocytes was significantly reduced in male and female ERα knockout animals compared to all wild-type and heterozygote animals (Fig. 6A). Mean podocyte volume was significantly enhanced in male and female ERα knockout mice compared to all other groups (Fig. 6B). Mesangial cell number and volume showed the same trends, which reached significance in several intergroup comparisons (Fig. 6A and 6B).

**Table 1:** **Glomerular number, volume and sclerosis index.** No parameters varied significantly between groups (WT =  wild type, Hetero  =  heterozygote, KO  =  knockout; n = 6 animals per group).

	Number of glomeruli (left kidney)	Mean glomerular volume [10^6^ µm^3^]	Glomerular sclerosis index
femaleWT	22142±4318	0.54±0.19	0.75±0.69
maleWT	25004±6986	0.33±0.12	0.87±0.37
femaleHetero	21788±7458	0.37±0.17	1.31±0.33
maleHetero	20499±7988	0.50±0.25	0.98±0.46
femaleKO	22694±4454	0.35±0.11	0.98±0.13
maleKO	20278±4555	0.34±0.07	0.97±0.50
ANOVA	p = 0.798 (n.s.)	p = 0.113 (n.s.)	p = 0.433 (n.s.)

Tgfβ1 and Collagen type IV expression were increased in ERα knockout animals compared to wild-type and heterozygous controls (Fig. 6C and 6D).

## Discussion

While clinical and epidemiological studies provide an increasing body of evidence for substantial gender differences regarding the progression of chronic glomerular diseases, this report demonstrates a direct protective effect of estradiol on podocyte survival.

The consistent demonstration of ERα expression in cultured murine podocytes and in podocytes of murine and human kidney specimens underlines the biological relevance of ERα for kidney physiology. In cultured podocytes, immunofluorescence staining showed ERα protein with cytoplasmic and nuclear localization. For these experiments, we used podocytes that were exposed to a phenol red-containing medium and non-stripped FCS, explaining a basal stimulation of ERα protein expression in both nuclear and cytoplasmatic compartments.

For a more detailed analysis, control cells for Western blot were cultured using phenol red-free medium and stripped FCS to exclude any residual estrogenic influences. This led to an ERα detection only in the nuclear compartment, while cytoplasmic protein did not reveal significant ERα expression. After estradiol stimulation, ERα was detected also in the cytoplasmic compartment, and expression was extensively enhanced in the nuclear compartment. Whether this is only due to an increased production of ER or indicates further relocation processes between nucleus and cytoplasm has not been addressed in detail.

Extensive studies are available for breast carcinoma cells, in which ER relocation between nucleus, cytoplasm and cell membrane was shown to be induced by estrogen, mediated by the Src homologous and collagen (Shc) adapter protein [23], which in turn associates with f-actin [24]. Interestingly, these adapter proteins and relocation processes turned out to be critical for many non-genomic estrogenic actions in breast-carcinoma cells, such as activation of mitogen-activated protein kinase (MAPK) and phosphoinositide 3-kinase (PI3-kinase) [25].

However, our results may point to similar interaction and relocation processes also in podocytes. However, further analyses are required to delineate the biological relevance of these findings.

As podocyte apoptosis represents one of the key mechanisms in glomerular damage, we were especially interested in the effects of estradiol on podocyte apoptosis. PAN treatment is the best-established model of apoptosis induction in podocytes and is regarded as a reliable experimental model for several glomerular diseases [26]. While animals treated with a single dose of PAN develop minimal-change disease-like transient proteinuria, repeated application results in persistent proteinuria and focal segmental glomerulosclerosis (FSGS), mimicking FSGS in humans.

Applying this model in-vitro, we were able to show significant prevention of PAN induced apoptosis by estradiol using two different methods of apoptosis detection. In the FACS analysis, the maximum percentage of apoptotic cells after PAN treatment showed some variation between different experiments. This may be explained by the high susceptibility of the method to even the slightest variations in the experimental procedure. Enzymatic detachment of cells from culture dishes, for example, is known to induce Annexin-FITC positivity in cells. Nevertheless, the reproducibility of the relative changes between treatment groups as well as the good correlation with the results obtained by Hoechst staining confirm the significance of our FACS results. Our results are supported by recent data by Doublier et al., who demonstrated that ER-mediated signalling protects podocytes from Tgfβ1 or TNFα-induced apoptosis, while testosterone induced podocyte apoptosis by signalling via androgen receptors [11].

Regarding the underlying mechanisms of estrogen action, we analyzed the mitochondrial membrane potential in podocytes. It is critical for the process of apoptosis via two distinct mechanisms. First, opening of the mitochondrial permeability transition pore (PTP) leads to the release of apoptogenic factors from the intermembrane space into the cytosol (e.g. cytochrome c, apoptosis inhibiting factor, Smac/Diablo). Second, changes of mitochondrial membrane potential cause disturbances in the process of oxidative phosphorylation, leading to cellular damage by impaired ATP/energy metabolism. These processes can occur either as an initiation step towards apoptosis or as a consequence of the apoptotic process, depending on the examined cellular system and the mechanism of damage induction [27]. Adenine nucleosides, such as PAN used in our experiments, disrupt mitochondrial PTP. Thus, changes in the mitochondrial membrane potential represent an early step of apoptosis in our experimental model.

In line with these assumptions, we demonstrate for the first time a PAN induced destabilization of mitochondrial membrane potential in podocytes. I was possible to reduce this effect by pretreatment with estradiol and even more by dexamethasone. Similar results were already investigated extensively in neurons and other cells, where estrogens show cytoprotective effects by interfering with mitochondrial function via nuclear and non-nuclear signalling pathways [19], [28].

Other possible mechanisms, such as activation of MAPK and inositol-triphosphate-kinase (IP3K) phosphorylation by membranous ER, were shown to have significant impacts on actin cytoskeleton, cellular proliferation, apoptosis and movement. For example, estrogen-induced activation of the Raf-MEK-ERK-p90RSK cascade was already shown to mediate protection of neurons against apoptosis via stabilization of Bad phosphorylation [21]. In our study, we demonstrated intensive stimulation of ERK1/2 phosphorylation induced by estradiol also in podocytes. This may contribute to the anti-apoptotic effect of estradiol in podocytes. Similar data supporting our hypothesis were obtained very recently by Doublier et al., who demonstrated a reduced antiapoptotic effect of estradiol in podocytes after ERK inhibition [11]. In contrast, estradiol hardly increased p38 MAPK phosphorylation, which is generally regarded as “stress-activated” kinase, promoting inflammation and apoptosis [29]. These findings underline the importance of non-genomic signalling for anti-apoptotic action of estradiol in podocytes.

It is noteworthy, that all our experiments analysing estrogen effects were performed with cell culture medium containing physiologic doses of estradiol before induction of apoptosis by PAN. Therefore, our results primarily focus on estrogenic influences on the prevention of injury and on the modification of the disease course. Further studies will have to address the possible therapeutic actions of estrogen for established renal injury using pharmacological doses of estradiol after induction of experimental damage. In addition, cyclic variation of estrogen concentration, as occurring *in-vivo*, may lead to different results.

To evaluate the *in-vivo* relevance of our data, we compared glomerular structures of ERα knockout mice to those of wild-type and heterozygous controls. In homozygous animals, podocytes are fewer, but bigger than in heterozygous and wild-type controls. Both findings – loss of podocyte number and cellular hypertrophy - indicate podocyte injury in ERα knockout animals.

Upregulation of Tgfβ1 and Tgfβ1-mediated collagen type IV expression in glomeruli of ERα knockout mice complemented the evidence of podocyte damage. Tgfβ1-expression is known to be associated with podocyte damage by the stimulation of extracellular matrix accumulation [30], while estrogen supplementation was shown to prevent Tgfβ1 mediated glomerulopathy [31]. The glomerular sclerosis index, another unspecific indicator of structural glomerular damage, also shows a trend towards increased sclerosis in knockout animals, but did not reach statistical significance after correcting with Tamhane's *post-hoc* analysis.

In ERα knock-out mice, proteinuria was shown to develop between 6 and 9 months of age [10]. To demonstrate early processes of podocyte damage before a more global glomerular damage and overt proteinuria has developed, we used 6 month-old mice and did not apply any further experimental renal damage. In contrast to other groups [10], [12], [32], we were able to demonstrate deleterious effects of ERα knockout not only in female but also in male animals. [10], [12], [32]. Thus, ERα-mediated signalling is critical in mice of both genders for maintaining healthy kidney function.

Whether the renal damage in ERα knockout animals is caused by the lack of podocyte ERα expression or by secondary effects due to the unspecific knockout of ERα throughout the body remains to be shown. The unselective ERα knockout leads to increased estrogen and luteinizing hormone (LH) levels via decreased negative feedback mechanisms through the hypothalamic-pituitary-gonadal axis [33]. The increased LH level induces 17beta-hydroxysteroid dehydrogenase type III activity in ovaries of ERα knockout mice, stimulating ovarian production of testosterone that further compromises renal outcome [10], [33]. Whereas our *in-vivo* data may also be explained by these secondary effects, our *in-vitro* data as well as recent data from other groups [11] support a protective role for the ERα expression in podocytes.

Interactions between gender hormones and kidney structure and function are probably even more complex. Although for cytoprotective action ERα seems to be of major interest [34], podocytes also express ERβ and androgen receptors [11]. Consequently, interactions between gender and podocyte biology may be based on an interplay between androgenic and estrogenic hormones, influencing a branched signalling network with various downstream effects, e.g. on proliferation, apoptosis, cytoskeleton and movement.

Finally, our data raise the question of a therapeutic applicability of estrogens for chronic glomerular diseases. There is one anecdotal report published in 1955, in which estrogens were used for the therapy of three patients (two women and one man) with nephrotic syndrome [35]. Initially, all three had complete remission of proteinuria, but unfortunately relapsed after the discontinuation of the therapy. The two women immediately received an additional course of hormones and again achieved remission, while the man could not be convinced to take more estrogens due to the significant side effects and sadly died in a nephrotic crisis.

The potential therapeutic use of estrogens for renal diseases has to be regarded with caution as hormone replacement therapy may be associated with increased risk of breast cancer and cardiovascular diseases [36]. Short-term side effects, such as feminization, may have substantial impact on the compliance of male patients. Furthermore, postmenopausal hormone replacement therapy (HRT) in women and experimental high-dose estrogen administration in mice have been shown to be associated with renal damage [37], [38]. Others demonstrate an improved renal prognosis for women under HRT [39]. However, selective estrogen receptor modulators (SERM) with tissue specific effects and less feminizing potential may provide a reasonable new option for patients with chronic glomerular diseases.

In conclusion, podocytes contain ERα which upon activation mitigates experimentally induced apoptosis in-vitro. The early stage of glomerulopathy in ERα knockout mice is characterized by a reduced number of podocytes and morphological changes, which are in line with an *in-vivo* relevance of ER for podocyte viability and function. Thus, our findings provide a novel model explaining protective influences of female gender on the outcome of chronic glomerular diseases in humans.

## Materials and Methods

### Cell culture

Immortalized murine podocytes were a generous gift of Peter Mundel (University of Miami, USA) and cultured as described previously [40]. Cells for analyzing estrogen influences were maintained in charcoal-stripped fetal bovine serum (FB-1001F/500, Biosera, East Sussex, UK) and phenol-red free culture medium (E15-848, PAA, Pasching, Austria) for at least 6 days before starting the experiments to exclude residual estrogenic action of culture medium.

Differentiation of cells was confirmed by ensuring arborized morphology and expression of *Synaptopodin* and *Wt1* with real-time PCR and Synaptopodin by immunocytochemistry (results not shown).

### Immunocytochemical/Immunohistochemical detection of ERα

For immunofluorescence, cells were grown on glass coverslips. After 14d of differentiation cells were fixed with PBS/4% paraformaldehyde and permeabilized with PBS/0.5% Triton X-100/3% BSA.

ER protein was visualized by indirect immunofluorescence using ERα antibody (C1355, Millipore, Schwalbach, Germany) at 1∶500 dilution for 12 h at 4°C, and chicken anti-rabbit secondary antibody coupled to Alexa fluor 594 (Molecular Probes, Oregon, USA) at 1∶1000 dilution for 60min at room temperature. All cells were co-stained with 4,6-diamidino-2-phenylindole dihydrochloride (DAPI, 1 µg/mL) for visualisation of cell nuclei. Coverslips were mounted on microscope slides; images were acquired with *Nikon Eclipse T_i_ inverted fluorescence microscope and NIS Elements AR 3.1 software (Nikon, Duesseldorf, Germany)* using a 20x objective lens. Negative controls were done without ERα primary antibody.

ERα staining of human kidney biopsy specimen was performed during routine histopathologic examination as described previously [41]. Breast and heart tissue specimens were used as positive and negative controls, respectively.

ERα staining of murine kidney tissue was carried out on paraffin sections using ERα antibody (sc-7207, Santa Cruz, Santa Cruz, USA) in 1∶25 dilution for 1 h at room temperature, followed by 3% H_2_O_2_ for 7 min. Primary antibody was detected using the *Histofine Simple Stain MAX-PO (Multi)* kit (Nichirei Co, Tokyo, Japan) and AEC substrate (DAKO, Hamburg, Germany) for 10 min. Color development was stopped by adding Aqua dest, finally sections were counterstained with hematoxylin.

### Nuclear-cytoplasmic fractionation, protein isolation and ERα Western blot

Cells were grown as described above, using phenol-red free medium and stripped FCS for at least 6 days before beginning of experiments. After 12 d of differentiation, cells were starved for 24 h with FCS concentration reduced to 1%. Cells dedicated for estrogen treatment were exposed to a medium containing 10 nM 1,3,5-Estratriene-3,17ß-diol (estradiol, E2758, Sigma-Aldrich, Munich, Germany), and controls were maintained in estrogen-free culture medium. Cells were detached after 24 h or 48 h, respectively, using Alfazyme (L11-012, PAA) for 5 min in 37°C. Afterwards, cells were pelleted and put on ice. Nuclear-cytoplasmic fractionation and protein extraction were conducted using the *NE-PER Nuclear and Cytoplasmic Extraction Reagents* kit (Thermo Scientific, Rockford, USA) according to the manufacturer's protocol.

For Western blot, 4x *NuPAGE LDS Sample Buffer* (Invitrogen, Darmstadt, Germany) was added to protein samples and boiled for 5 min at 72°C. Proteins were separated using *NuPAGE Novex 12% Bis-Tris Gel* (Invitrogen) and transferred on *Immobilon-P PVDF membrane* (Millipore) using a *XCell SureLock Mini-Cell* electrophoresis and blotting system (Invitrogen). *SeeBlue Plus2 prestained Standard* (Invitrogen) was used as a protein marker.

The membrane was blocked in 5% milk for 1h at room temperature and incubated overnight at 4°C with ERα antibody (ab2746, Abcam, Cambridge, UK), diluted 1∶1000 in *Super Block Blocking Buffer* (Thermo Scientific). After washing with PBS and incubation with horseradish peroxidase-conjugated anti-mouse IgG (JL315035044, Jackson Immuno Research, Suffolk, UK), detection was performed by the *Super Signal West Pico Chemiluminescent substrate* (34080, Thermo Scientific) and visualized by *Fujifilm Super-RX* Film (47410 08389, Fujifilm, Duesseldorf, Germany). Film was processed using C*urix 60* film processor (AGFA, Greenville, USA) and scanned with a *Canon SmartBase MPC600F* scanner (Canon, Krefeld, Germany).

For differentiation between protein fractions, anti-GAPDH antibody (sc-48166, Santa Cruz, Santa Cruz, USA) was used as marker for cytoplasmatic fraction, diluted 1∶3000 in *Super Block Blocking Buffer* (Thermo Scientific) and incubated overnight at 4°C. The nuclear fraction was detected by anti-Phospho-Histone H3 antibody (#9714, Cell Signaling, Danvers, USA; diluted 1∶1000 in *Super Block Blocking Buffer,* and incubated overnight at 4°C).

### Apoptosis induction and detection in cultured podocytes

After 14d of differentiation, cells dedicated for estradiol treatment were pretreated by culture medium with 10 nM estradiol for 24 h. Control groups were exposed to medium change only. Apoptosis was induced by a medium with 30 µg/ml Puromycin aminonucleoside (PAN, P7130, Sigma-Aldrich) for 48h in the presence or absence of estradiol. As positive control, cells were treated with 1 µM (11β,16α)-9-Fluoro-11,17,21-trihydroxy-16-methylpregna-1,4-diene-3,20-dione (dexamethasone, D4902, Sigma-Aldrich) in the same time-intervals and treatment groups as estradiol. Apoptosis was quantified by the following methods:

*Hoechst 33342 Staining:* After collecting supernatant medium with detached cells, cultures were washed with PBS and detached using Alphazyme (L11-012, PAA) for 8 min at 37°C, pelleted for 5 min by centrifugation at 1100 rpm, washed with PBS, resuspended in 2.5 ml native medium and incubated for 1h at 37°C. After that, *Hoechst 33342* (14533, Sigma-Aldrich) was added to the medium to a final concentration of 1 µM/ml for 15 min at 37°C. Cells were fixed for 15min at room temperature with 4% formalin (6459124, Fischar, Saarbruecken, Germany). After pelleting, cells were resuspended in 100 µl PBS, dispensed on a microscope slide and dried for 1h in the dark. Slides were covered by *Prolong Gold Antifade reagent* (P36930, Invitrogen) and coverslips. Cells were visualized by an *Axiovert 200M fluorescence microscope* (Zeiss, Oberkochen, Germany). Apoptosis was defined as the percentage of cells showing nuclear condensation/fragmentation in at least 300 consecutive cells.

*Flow cytometry:* Apoptosis was assessed using *Annexin V-FITC kit* (PN IM3546, Beckman Coulter, Krefeld, Germany). Cells were harvested from culture flasks as described above. After 1h of incubation in 10ml native culture medium, cells were pelleted and resuspended in *Annexin binding buffer*. The following staining protocol was performed according to the manufacturer. Staining was detected on a *FACSCalibur* flow cytometer (Becton Dickinson, Heidelberg, Germany). At least 10,000 cells per treatment condition were analyzed on *CellQuest Pro* software (Becton Dickinson, Heidelberg, Germany). Cells that were Annexin V positive and propidium iodide negative were considered apoptotic.

### TMRM-staining of mitochondrial membrane potential

After 14d of differentiation on glass coverslips, apoptosis was induced with PAN (30 µg/ml) for 48h in the presence or absence of 10 nM estradiol. After 48 h, podocytes were incubated in a medium containing 100 nM tetramethyl rhodamine methyl ester (TMRM; Invitrogen) for 25 min at 37°C, allowing mitochondria to load with TMRM fluorescent dye in proportion to mitochondrial membrane potential. After washing with PBS, coverslips were mounted in chambers containing HEPES-Tris buffer (pH 7.4, prewarmed at 37°C). The chambers were mounted on an *Axiovert 200M fluorescence microscope* (Zeiss) equipped with a 63x/1.4 oil immersion objective. Life-cell images were acquired using *Axiovision* software. Further image processing and analysis were performed using *Image Pro* software (Media Cybernetics, Silver Spring, MD, USA) as described before in detail [42]. Staining intensity is given in arbitrary units.

### ERK1/2 and p38 MAPK Western Blot

Cells were grown as described above, using phenol-red free medium and stripped FCS for at least 6 days before beginning of experiments. After 12d of differentiation, cells were starved for 24 h with FCS concentration reduced to 1%. Cells dedicated for estrogen treatment were exposed to medium containing 10nM estradiol, controls were maintained in estrogen-free culture medium.

Cells were harvested after 0, 1, and 5 minutes using *Tissue Protein Extraction buffer* (#78510, Thermo Scientific) supplemented with *Complete Protease Inhibitor Cocktail* (Roche, Indianapolis, USA), incubated on ice for 10 min, centrifuged for 10 minutes at 4°C and 16,000 g. Supernatant was collected and supplemented with *4x NuPAGE LDS Sample Buffer (Invitrogen)*. Lysates were boiled for 10 minutes at 72°C and loaded onto *NuPAGE 4-12% Bis-Tris Gel* (Invitrogen). Protein separation, blotting and membrane blocking were performed as described above.

Stainings were performed using anti-phospho ERK1/2 (#9101, Cell Signaling, 1∶1,000), anti-ERK1/2 (#9102, Cell Signaling, 1∶1,000), anti phospho-p38 MAPK (#9215, Cell Signaling, 1∶1,000) and anti-βActin antibodies (A5441, Sigma-Aldrich, 1∶3,000), each diluted in *Super Block Blocking Buffer* (Thermo Scientific) and incubated overnight at 4°C.

After washing, the membrane was incubated with horseradish peroxidase-conjugated anti-rabbit IgG (JL711035152, Jackson Immuno Research, 1∶10,000; against phospho-ERK1/2, ERK1/2, phospho-p38 MAPK) and anti-mouse IgG (JL315035044, Jackson Immuno Research, 1∶20,000; against β-Actin) for 1 hour at room temperature.

Signal detection was performed by the *Super Signal West Pico Chemiluminescent substrate* (34080, Thermo Scientific) and visualized as described above.

### ERα knockout mice

All animal experiments were approved by the Institutional Animal Care and Use Committee of the University Hospital Heidelberg (permit numbers AZ 35-9185.81/G-103/02 and AZ 35-9185.81/G-142/03) and conducted in accord with accepted standards of humane animal care. All efforts were made to minimize suffering.

We used ERα^-/-^ mice generated from C57BL/6 mice as previously described by Dupont et al. [43], kindly provided by Prof. Pierre Chambon (Strasbourg, France) and Dr. Theo Pelzer (Wuerzburg, Germany). Mice were housed in box cages under standard conditions and bred to yield homozygous (ERα^-/-^), heterozygous (ERα^+/-^) and wild-type (ERα^+/+^) animals. The correct genotype was confirmed by PCR amplification of genomic DNA extracted from tail tissue. All animals had free access to water and chow (Altromin 1324, 1% NaCl, soy-free, low in phytoestrogens; Altromin, Lage, Germany).

Six months old mice were allocated to the following experimental groups: (1) wild-type females, (2) heterozygous females, (3) homozygous knockout females, (4) wild-type males, (5) heterozygous males, (6) homozygous knockout males; each group contained n = 6 animals.

Animals were sacrificed after anaesthesia with ketamine and xylazine. After perfusion with NaCl 0,9%, right kidney was harvested; 50% was frozen in liquid nitrogen, the remaining tissue formaline-fixed for immunohistochemical staining.

Then the remaining animal was perfused with 3% phosphate-buffered glutaric aldehyde before harvesting of the remaining organs. The left kidney was sectioned in a plane perpendicular to the interpolar axis, yielding slices of 1–2 mm width. Five representative samples of renal cortex were cut using area weighted sampling [44] with subsequent additional fixation with glutaric aldehyde for 4 h, washing with PBS and another fixation step in 1% osmium tetroxide for 1h. Semithin sections of 1 µm were prepared and stained with periodic Schiff-acid for analysis of glomerular sclerosis index/glomerular number or stained with toluidine blue for analysis of podocyte/mesangial cell number and cell volume.

The glomerular sclerosis index, indicating glomerular damage, was calculated as described previously [45]. In five semithin sections for each animal, the glomerular number and volume were analyzed as described previously [46], [47]. Glomerular cell numbers and cell volumes (podocytes and mesangial cells) were calculated as described previously in at least 30 glomeruli per animal [46], [48].

Immunohistochemical stainings of Tgfβ1 and Collagen type IV were performed with paraffin sections as described previously [49], using the following antibodies: anti-Tgfβ1 (1∶100 dilution for 1h at room temperature; Santa Cruz), anti-Collagen type IV antibody (1∶100 dilution for 1h at room temperature; Biotrend, Cologne, Germany). Stainings were analyzed by two blinded independent investigators; scoring was performed semiquantitatively in 20 glomeruli per kidney as described elsewhere [46].

### Statistical analysis

Results are given as mean ±SE. Statistical analysis was performed using SPSS 18.0 software for Mac (SPSS Inc., Munich, Germany) and one-way ANOVA with Tamhane post-hoc analysis. Statistical significance was defined as p<0.05.
